# Identification of plexin A4 as a novel clusterin receptor links two Alzheimer’s disease risk genes

**DOI:** 10.1093/hmg/ddw188

**Published:** 2016-07-04

**Authors:** Silvia S. Kang, Aishe Kurti, Aleksandra Wojtas, Kelsey E. Baker, Chia-Chen Liu, Takahisa Kanekiyo, Yuetiva Deming, Carlos Cruchaga, Steven Estus, Guojun Bu, John D. Fryer

**Affiliations:** 1 Department of Neuroscience, Mayo Clinic, Jacksonville, FL, SA; 2 Neurobiology of Disease Graduate Program, Mayo Clinic College of Medicine, Jacksonville, FL; 3 Department of Psychiatry and Hope Center, Washington University School of Medicine, St. Louis, MO, USA; 4 Sanders-Brown Center on Aging, Department of Physiology, University of Kentucky, Lexington, KY, USA

## Abstract

Although abundant genetic and biochemical evidence strongly links Clusterin (CLU) to Alzheimer disease (AD) pathogenesis, the receptor for CLU within the adult brain is currently unknown. Using unbiased approaches, we identified Plexin A4 (PLXNA4) as a novel, high-affinity receptor for CLU in the adult brain. PLXNA4 protein expression was high in brain with much lower levels in peripheral organs. CLU protein levels were significantly elevated in the cerebrospinal fluid (CSF) of *Plxna4^-/-^* mice and, in humans, CSF levels of CLU were also associated with *PLXNA4* genotype. Human AD brains had significantly increased the levels of CLU protein but decreased levels of PLXNA4 by ∼50%. To determine whether PLXNA4 levels influenced cognition, we analyzed the behaviour of *Plxna4^+/+^*, *Plxna4^+/-^*, and *Plxna4^-/-^* mice. In comparison to WT controls, both *Plxna4^+/-^* and *Plxna4^-/-^* mice were hyperactive in the open field assay while *Plxna4^-/-^* mice displayed a hyper-exploratory (low-anxiety phenotype) in the elevated plus maze. Importantly, both *Plxna4^+/-^* and *Plxna4^-/-^* mice displayed prominent deficits in learning and memory in the contextual fear-conditioning paradigm. Thus, even a 50% reduction in the level of PLXNA4 is sufficient to cause memory impairments, raising the possibility that memory problems seen in AD patients could be due to reductions in the level of PLXNA4. Both *CLU* and *PLXNA4* have been genetically associated with AD risk and our data thus provide a direct relationship between two AD risk genes. Our data suggest that increasing the levels of PLXNA4 or targeting CLU-PLXNA4 interactions may have therapeutic value in AD.

## Introduction

Alzheimer disease (AD) is the most common cause of dementia and affects >10% of individuals over the age of 65. Hallmark features of AD pathology include extracellular amyloid plaque formation and intracellular tau accumulation in the central nervous system (CNS). Both non-fibrillar (diffuse) and fibrillar (true “amyloid”) plaques are evident in AD, with the latter being highly associated with toxicity and neuritic dystrophy *in vivo*. The ϵ4 allele of the apolipoprotein E (APOE) gene is the strongest AD genetic risk factor. Interestingly, Clusterin (CLU), also known as Apolipoprotein J (ApoJ), shares many overlapping molecular functions with APOE including direct binding to amyloid β (Aβ) *in vivo* and *in vitro* and regulation of Aβ pathology (1). Previous studies have shown that CLU is critical for fibrillar amyloid plaque induced neuritic dystrophy *in vivo* (2), a presumed mechanism for neuronal dysfunction. Additionally, *CLU* variants were significantly associated with AD risk in multiple large human genetic association studies (3,4).

Although CLU has high brain expression and impacts aspects of AD pathology, relatively little is known regarding CLU receptor biology in the CNS. The most recognized CLU receptor, low-density lipoprotein receptor related protein 2 (LRP2, also known as Megalin), is not present in the adult CNS with the exception of ependymal cells (5). Other recently reported CLU receptors such as low-density lipoprotein receptor related protein 8 (LRP8, also known as APOER2) and very low-density lipoprotein receptor (VLDLR) (6) have not been proven to function as CLU receptors *in vivo*. We therefore hypothesized that an additional, yet to be defined, receptor for CLU exists in the CNS and may be involved in mediating AD pathogenesis.

Here, we demonstrate that Plexin A4 (PLXNA4) is a novel, high affinity receptor for CLU in the adult CNS. The expression of PLXNA4 influences the levels of CLU in the cerebrospinal fluid (CSF) in both mice and humans. PLXNA4 is highly expressed in brain tissue and is significantly reduced in brain lysates from the patients with AD compared to controls. Finally, the analysis of *Plxna4^+/-^* and *Plxna4^-/-^* mice demonstrates that reduction or loss of PLXNA4 results several behavioural impairments. Given the recent genetic association of the *PLXNA4* gene with AD risk (7), our data suggest that the CLU-PLXNA4 signalling pathway could represent a viable and important therapeutic target for AD.

## Results

**Figure 1.**
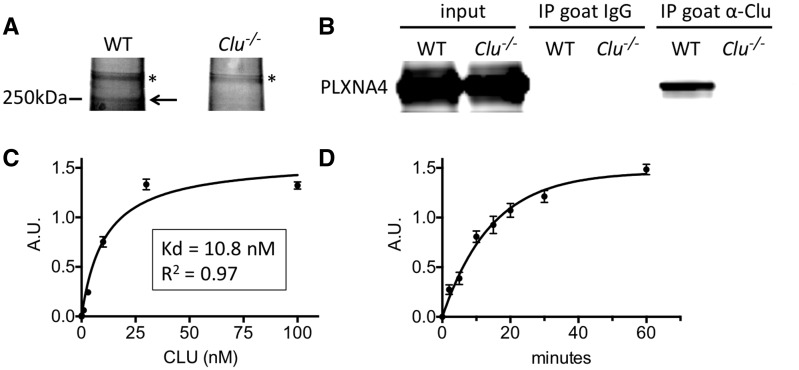
CLU binds directly to PLXNA4 *in vivo* and *in vitro*. (**A**) Silver stained gel showing a specific band (arrow) immunoprecipitated with anti-CLU antibody in WT but not *Clu^-/-^* brain lysates (* indicates non-specific band). The specific CLU-interacting band was cut out from the gel and identified as PLXNA4 by mass spectrometry. Image shown is representative of > 3 experimental replicates. (**B**) Immunoprecipitation for CLU from brain lysates followed by Western blotting for PLXNA4 demonstrates specific interaction between CLU and PLXNA4 in WT mice but not *Clu^-/-^* mice or when using non-specific goat IgG as control. Image shown is representative of > 3 experimental replicates. (**C**) Cell-free, solid phase binding assay with immobilized PLXNA4 incubated with increasing amounts of CLU showing high affinity (K_d _=_ _10.8 nM), saturable binding (average of three independent experiments). (**D**) Time-course of CLU binding to immobilized PLXNA4 demonstrates that the CLU-PLXNA4 interaction occurs very rapidly with half-maximal binding at 10.03 min. A.U. = arbitrary units.

To discover novel CLU receptors in the adult brain, CLU protein was immunoprecipitated (IP’d) from wild-type (WT) or *Clu^-/-^* mouse brains, providing an ideal negative control for CLU-binding specificity. Silver stain analysis of proteins that co-IP’d with CLU consistently yielded a ∼250 kDa band in WT but not *Clu^-/-^* brain samples (Figure 1A). This band was excised and identified by mass spectrometry as mouse PLXNA4 (protein ID# Q80UG2). To validate CLU association with this potential receptor, IP for CLU followed by Western blotting to probe for PLXNA4 was conducted. PLXNA4 specifically co-IP’d with CLU from WT brain with no detection in negative controls that utilized *Clu^-/-^* lysates or normal goat IgG (Figure 1B). To determine whether CLU directly associated with PLXNA4, we performed solid phase binding assays with immobilized human PLXNA4 and increasing amounts of human CLU protein. CLU binding to PLXNA4 occurred in a direct, high-affinity manner with a Kd of 10.8 nM (average of three independent experiments, Figure 1C). Interestingly, this is a slightly higher affinity than the reported Kd of CLU for LRP2/Megalin under similar conditions (Kd = 14.2 nM) (8). The saturable binding nature demonstrates that this is a specific association. We found that CLU bound to PLXNA4 very rapidly in this assay with half-maximal binding at 10 min (Figure 1D).

PLXNA4 has known roles in the developing nervous system (9–11), but very little data exists on PLXNA4 in adult organisms. To further study PLXNA4 in the brain, we obtained *Plxna4^-/-^* mice on a C57BL/6J background (10). We bred *Plxna4^+/-^* males to *Plxna4^+/-^* females to derive each of the possible genotypes: *Plxna4^+/+ ^*“wild-type” (WT), *Plxna4^+/-^* (heterozygous), and *Plxna4^-/-^* (knockout) mice. We first tested whether commercially available antibodies were able to specifically recognize endogenous PLXNA4 from brain tissue lysates. We identified several antibodies from rabbit, mouse and sheep that detect a single clean band in WT mice but not in *Plxna4^-/-^* mice (Supplementary Material, Fig. S1). Human and mouse PLXNA4 are 97.7% identical at the amino acid level across the entire protein and are 100% identical at the epitopes of these antibodies. Importantly, these results also demonstrate that *Plxna4^+/-^* mice have a ∼50% reduction in the amount of PLXNA4 protein compared to WT mice (Supplementary Material, Fig. S1).

**Figure 2.**
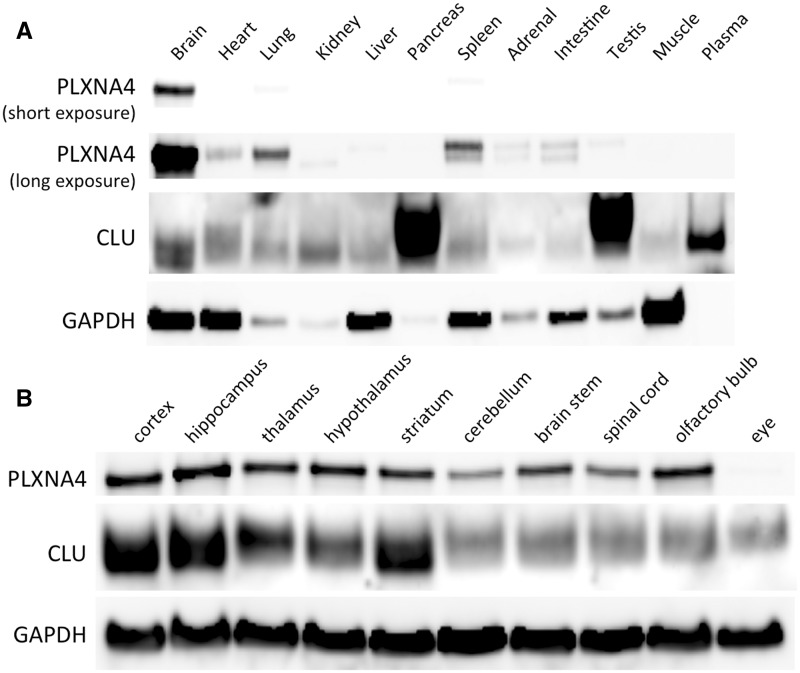
Expression of PLXNA4 and CLU in various tissues by Western blotting. (**A**) PLXNA4 is highly expressed in brain tissue. Longer exposures show expression of PLXNA4 in lung and spleen, with low but detectable levels in heart, kidney, liver, adrenal, intestine, and testis. No detectable levels were found in pancreas, muscle, or plasma. Slight differences in molecular weights of PLXNA4 are likely due to post-translational modifications. CLU is broadly expressed throughout the body, with highest expression in pancreas, testis, and plasma. CLU is easily detectable in all tissues surveyed, including whole brain tissue, with some tissues also showing differences in molecular weights due to the known heavily glycosylated nature of CLU. (**B**) Both PLXNA4 and CLU are highly expressed throughout all regions of the central nervous system (with the exception of lack of PLXNA4 in the eye). Highest expression of both PLXNA4 and CLU is seen in the hippocampus.

We next surveyed several organs by Western blotting and found extremely high expression of PLXNA4 in the adult brain of WT mice with much lower levels in lung and spleen (Figure 2A). Longer exposures demonstrated low but detectable levels of PLXNA4 in the heart, kidney, liver, adrenal, intestine, and testis but no readily detectable signal in pancreas, muscle, or plasma (Figure 2A). Within the CNS, PLXNA4 was broadly expressed in all brain regions with somewhat lower levels in the cerebellum and spinal cord but not the eye while CLU was also broadly expressed in all brain regions (Figure 2B). Within the CNS, the levels of both PLXNA4 and CLU were highest in the hippocampus.

**Figure 3.**
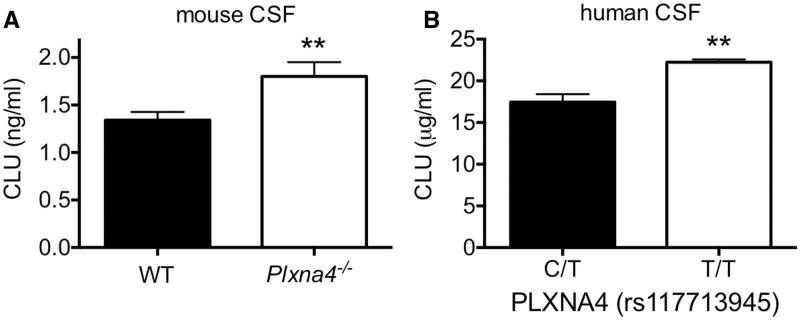
PLXNA4 is associated with levels of CLU in the cerebrospinal fluid (CSF) in mice and humans. (**A**) CSF isolated from WT (N = 14) and *Plxna4^-/-^* (N = 10) littermate mice was assayed for CLU protein levels by a sensitive sandwich ELISA. CLU was significantly elevated in *Plxna4^-/-^* mice by ∼34%. Data analyzed by two-tailed t-test, ** indicates *P <* 0.01. (**B**) CLU protein is significantly elevated by ∼27% in the CSF of individuals with the T/T allele (*n =* 23) compared to C/T allele (*n =* 631) at rs117713945 of *PLXNA4*. ** indicates *P <* 0.01 (Bonferroni corrected).

To assess whether the presence of PLXNA4 modulated CLU levels *in vivo*, we analyzed the cerebrospinal fluid (CSF), an easily measureable extracellular fluid of the brain, of WT and *Plxna4^-/-^* mice. CLU protein was significantly elevated by ∼34% in the CSF of *Plxna4^-/-^* mice compared to WT littermates (Figure 3A). To determine whether PLXNA4 could modulate CLU levels in humans, we analyzed the CSF of individuals that had been genotyped for PLXNA4 variants. We found that CLU was significantly elevated in the CSF of individuals with T/T alleles at rs117713945 of the *PLXNA4* locus (Figure 3B). These data demonstrate that the level of PLXNA4 significantly impacts CSF levels of CLU *in vivo* in mice and that *PLXNA4* is genetically associated with CLU levels in the CSF of humans.

**Figure 4.**
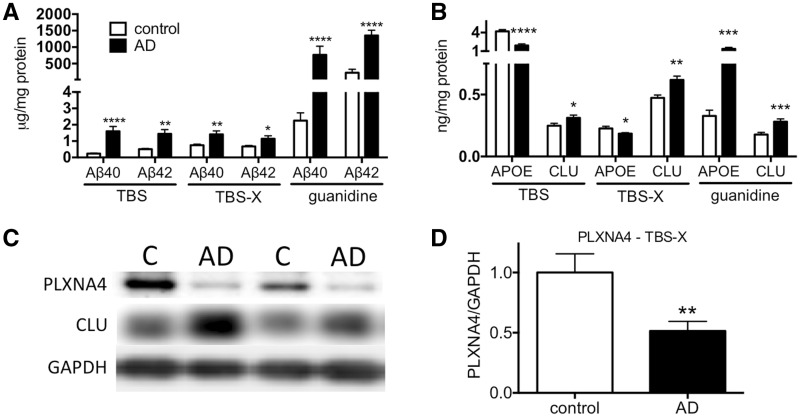
Alterations in CLU and PLXNA4 protein levels in Alzheimer’s disease brain tissue. (**A**) Aβ40 and Aβ42 are significantly elevated in the brains of AD cases compared to controls. (**B**) ApoE is significantly decreased in TBS and TBS-X fractions in AD cases but increased in guanidine fractions. In contrast, CLU is significantly elevated in TBS, TBS-X and guanidine fractions. (**C**) Representative Western blot of control vs AD showing substantial decrease of PLXNA4 and increase in CLU in AD cases. (**D**) PLXNA4 levels were significantly decreased in AD cases vs controls by ∼50%. N = 20 cases and N = 20 controls for A, B, and D, analyzed by two-tailed *t*-test.

To determine the relationship between CLU and PLXNA4 during AD pathology, we examined the expression of both proteins in human AD brain tissue lysates that have abundant amyloid accumulation. Temporal cortex from control and AD brains were sequentially lysed in TBS, TBS-X and guanidine. As expected, AD brain tissue had significantly elevated levels of Aβ_40_ and Aβ_42_ in all extracts (Figure 4A). The level CLU was also significantly elevated in soluble pools (TBS and TBS-X) as well as guanidine fractions, indicative of co-deposition of CLU with amyloid (Figure 4B). In contrast, apoE levels were lower in soluble pools, but elevated in guanidine fractions (Figure 4B). We next assayed PLXNA4 levels by Western blotting of the TBS-X fraction that contains the majority of membrane-bound receptors. In contrast to increased CLU levels in the TBS-X fraction, PLXNA4 levels were significantly decreased in AD cases compared to controls by ∼50% (Figure 4C, D).

The significant reduction of PLXNA4 levels in human AD brains prompted us to consider whether this receptor may play a role in the cognitive impairments seen in AD. To test this, we prepared a large cohort of male and female littermate mice that were *Plxna4^+/+ ^*“wild-type” (WT), *Plxna4^+/-^* (heterozygous), and *Plxna4^-/-^* (knockout) mice. We then performed a behavioural battery on these mice to determine whether PLXNA4 levels impacted activity, anxiety or learning and memory.

**Figure 5.**
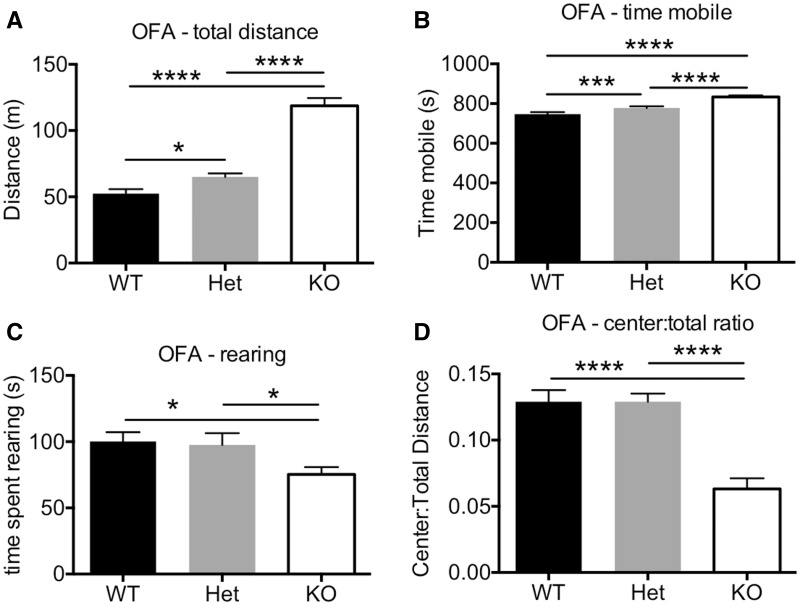
Behavioural alterations in the open field assay (OFA) in mice due to reduction or loss of PLXNA4. *Plxna4^+/-^* and *Plxna4^-/-^* mice were significantly more active than their WT littermates in the OFA by total distance travelled (**A**) and time spent mobile (**B**). However, only *Plxna4^-/-^* mice had alterations in rearing (**C**) and anxiety-like behaviour as measured by centre:total distance ratio (**D**) compared to WT littermates while and *Plxna4^+/-^* mice were apparently normal. Data analyzed by one-way ANOVA with post-hoc Fisher’s LSD *t*-test. N = 22/genotype of males and females combined. * *P <* 0.05, *** *P <* 0.001, **** *P <* 0.0001.

**Figure 6.**
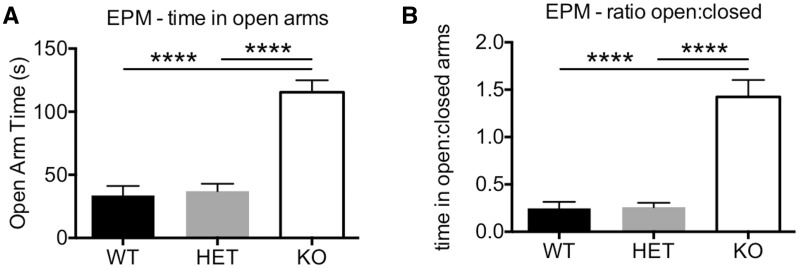
Loss of PLXNA4 in mice results in hyper-exploratory behaviour in the elevated plus maze (EPM). Compared to WT littermate controls, *Plxna4^-/-^* mice displayed significantly greater exploratory behaviour in the EPM as measured by time spent in the open arms (**A**) or as a ratio of the time spent in the open arms to the time spent in the closed arms (**B**). However, *Plxna4^+/-^* mice were apparently normal and were not significantly different that their WT littermate controls. Data analyzed by one-way ANOVA with post-hoc Fisher’s LSD *t*-test. N = 22/genotype of males and females combined. **** *P <* 0.0001.

Behavioural analysis of these mice in the open field assay revealed that reduction (*Plxna4^+/-^* mice) or loss (*Plxna4^-/-^* mice) of PLXNA4 resulted in a significant increase in overall activity evident by measures of total distance travelled (Figure 5A) and time mobile (Figure 5B). Additionally, rearing behaviour was also significantly decreased in *Plxna4^-/-^* mice but was normal in *Plxna4^+/-^* mice (Figure 5C). Apparent anxiety-like behaviour was also seen in *Plxna4^-/-^* mice, but not *Plxna4^+/-^* mice, as measured by the distanced travelled in the imaginary centre region normalized to the total distance travelled (Figure 5D). Additional testing of anxiety-like behaviour in the elevated plus maze demonstrated that *Plxna4^-/-^* mice, but not *Plxna4^+/-^* mice, actually exhibit lower anxiety-like behaviour than WT controls as shown by time in the open arms (Figure 6A) or the ratio of the time spent in the open arms to closed arms (Figure 6B). These data are somewhat surprising, but we have previously seen lower centre:total ratios in the open field assay (i.e. higher anxiety-like behaviour) but increased time in the open arms in the elevated plus maze (i.e. lower anxiety-like behaviour) in mouse models of neurodegeneration (12,13). These anxiety-like behaviours were not found in *Plxna4^+/-^* mice, suggesting that much larger reduction in PLXNA4 are necessary to observe these phenotypes or that complete absence of PLXNA4 during development causes these behaviours.

**Figure 7.**
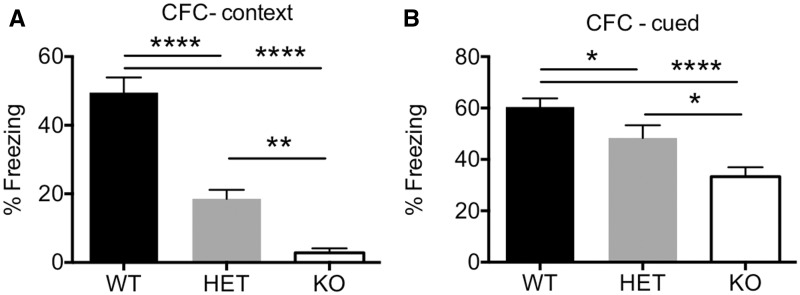
Reduction or loss of PLXNA4 in mice results severe memory impairments in the contextual fear conditioning (CFC) paradigm. Compared to WT littermate controls, both *Plxna4^+/-^ and Plxna4^-/-^* mice had significant memory impairments in the hippocampus-dependent contextual phase of the CFC (**A**) as well as the hippocampus/amygdala-dependent cued phase of the CFC (**B**). Data analyzed by one-way ANOVA with post-hoc Fisher’s LSD *t*-test. N = 22/genotype of males and females combined. * *P <* 0.05, ** *P <* 0.01, **** *P <* 0.0001.

We next analyzed these mice in the contextual fear-conditioning assay of associative learning and memory. We found that, compared to WT control mice, *Plxna4^-/-^* mice were severely impaired in both the hippocampus-dependent context portion of this assay (Figure 7A) as well as the hippocampus/amygdala-dependent cued portion of this assay (Figure 7B). Moreover, *Plxna4^+/-^* mice also showed learning and memory impairments in both the context (Figure 7A) and cued (Figure 7B) phases of the test, demonstrating that a 50% reduction in the amount of PLXNA4 is sufficient to cause significant cognitive deficits. Reduced freezing behaviour in general could contribute or confound the contextual and cued fear conditioning experiments. However, we found that baseline freezing in the training phase of the assay (before tone or foot shock) as well as the first 3 min of the cued phase of the assay (before the tone) was not different between genotypes (Supplementary Material, Fig. S2). Thus, it is unlikely that the memory impairments seen in *Plxna4^+/-^* and *Plxna4^-/-^* mice are due to artefacts of generalized freezing.

## Discussion

In summary, we have discovered that PLXNA4 is a novel, high affinity receptor for CLU in the adult CNS. We further demonstrated that PLXNA4 modulates CSF levels of CLU protein *in vivo* in both mice and humans. PLXNA4 is highly expressed in the brain and is substantially reduced in human AD brain samples compared to controls. Notably, reduction or complete loss of PLXNA4 in mice results in several behavioural phenotypes, including impairments in learning and memory.

Although CLU is an Aβ binding protein (14) and has been shown to be associated with AD pathology, there has been little focus directed at the role of CLU in AD and determining its receptor in the adult CNS. Human genetic association studies demonstrated that *CLU* variants are significantly associated with AD risk (3,4) and that the minor protective *T* allele (rs11136000T) of *CLU* is associated with increased transcript levels. These data suggest that elevated CLU may play a neuroprotective role in AD (15–17). Interestingly, to date, only one mouse model of AD amyloidosis, the PDAPP model (18), has been crossed to *Clu^-/-^* mice to determine how CLU shapes disease pathology. In contrast to what is suggested from human data, PDAPP *Clu^-/-^* mice show reductions in plaque load and neuritic dystrophy (2), suggesting a pathological role *in vivo*. At face value, the data from PDAPP; *Clu^-/-^* mouse suggesting decreased CLU is beneficial (i.e. less fibrillar plaque and less neuritic dystrophy) (1,2) is at odds with human data suggesting that increased *CLU* transcript observed in the minor protective *T* allele (rs11136000T), is beneficial. However, it is possible that a lifetime of mildly elevated CLU protein is beneficial and protective from AD development through its ability to bind Aβ, theoretically leading to increased Aβ clearance. Future work aimed at understanding the mechanisms by which CLU influences AD pathology would therefore also require knowledge of the CLU binding receptors and potential signalling pathways that may affect disease.

LRP2, a well-known receptor for CLU, is expressed during embryogenesis; however, LRP2 expression is no longer observed in the adult CNS, with the exception of ependymal cells. We discovered that PLXNA4 acts as a receptor for CLU and binds with a high affinity that rivals, if not exceeds, what has been shown for CLU-LRP2 interactions (Figure 1C). It is expressed throughout the brain, with highest expression in the hippocampus (Figure 3). Interestingly, *PLXNA4* was recently shown to be genetically associated with AD risk and those data indicated that PLXNA4 transcript levels are increased in AD brains (7). However, we demonstrated that PLXNA4 protein levels in human AD patients were significantly decreased relative to controls (Figure 5D). Taken together with the previous finding of increased PLXNA4 transcript levels in AD patients (7), these data suggest that neurons may be attempting to compensate for the loss of PLXNA4 protein by upregulating transcription.

One of the major aspects of AD is the cognitive decline in patients. Our behavioural analysis of *Plxna4^+/-^* and *Plxna4^+/-^* mice demonstrates that a reduction or loss of PLXNA4 is sufficient to cause behavioural impairments including cognitive deficits. Notably, PLXNA4 expression is highest in the hippocampus (Figure 3), a region that is known to impact learning and memory. Future studies of spatial learning and memory (e.g. water maze or Barnes maze) could help further delineate the role of PLXNA4 beyond the associative memory as shown here. This and other data are needed such as determining the timing of PLXNA4 reduction during the course of AD pathogenesis, but our data suggest that loss of PLXNA4 alone could potentially contribute to the memory problems or other behavioural manifestations that occur in this disease.

Currently, PLXNA4-mediated intracellular signalling pathways in the adult brain induced in general, or specifically by CLU-PLXNA4 interactions, are completely unknown. During development PLXNA4 is important for growth cone function and axonal guidance (10,11). Plexin receptor family members can bind semaphorins and function together with co-receptors such as neuropilins to guide many developmental processes; however, diverse or even opposing functions are observed depending on the particular context (19). Both *Clu^-/-^* and *Plxna4^-/-^* single knockout mice are perfectly viable. However, we have been unable to generate *Clu^-/-^; Plxna4^-/-^* double knockout mice although more than seven were expected from 115 pups born to date (p < 0.001, data not shown). Therefore, although the signalling is unknown, this “synthetic lethal” experiment indicates that the CLU-PLXNA4 pathway is critical during development.

Together, our data indicate PLXNA4 is a novel receptor for CLU and that therapeutics that increase PLXNA4 levels, modify activation state, or that enhance CLU-PLXNA4 binding could be used in the treatment of AD. Determining both CLU-dependent as well as CLU-independent regulation of the PLXNA4 pathway will be important in determining how this could be exploited as a therapeutic target.

## Materials and Methods

### Biochemical analysis of human samples

Temporal lobe cortex samples from neurologically unimpaired (*n =* 20) and AD (*n =* 20) subjects were obtained from the University of Kentucky Alzheimer’s Disease Centre. Diagnosis of AD followed clinical criteria and confirmed pathologically with an average Braak stage of 0-2 for controls and 5-6 for AD subjects. The average age of subjects was 84.2 ± 4.1 years for controls and 85.1 ± 5.7 years for AD subjects. Average postmortem interval was 3.2 h and roughly equal numbers of males and females were in each group.

### Genetic association of CLU levels in CSF from human samples

The Washington University Clinical Core evaluated a total of 654 individuals from the Knight Alzheimer’s Disease Research Center (ADRC). Cases were diagnosed in accordance with standard criteria and dementia severity was determined using the Clinical Dementia Rating. Rules Based Medicine, Inc used the Human Discovery Multi-Analyte Profile (MAP) panel to measure CLU levels. Analytes were used with a call rate at 90% or above (CLU had a call rate above 97.8%). Samples were genotyped with the Illumina 610 or Omniexpress chip. Minimum call rate for SNPs and individuals was 98% with 1,690 SNPs located in PLXNA4. We used an additive model in PLINK to test for association with normalized CLU protein levels in the CSF. Covariates were site, age, gender, and two principal component factors to adjust for population structure.

### Mice

A *Clu^-/-^* mouse colony (20) was established after cryorecovery from frozen embryos (Jackson Labs). A *Plxna4^-/-^* mouse colony was generated in-house using frozen sperm from previously published *Plxna4^-/-^* mice (10). All mice were on a pure C57BL/6J background. All studies were done in accordance with National Institutes of Health Guide for the Care and Use of Laboratory Animals under an approved protocol from the Mayo Clinic Institutional Animal Care and Use Committee.

### Tissue processing

Human postmortem brain tissues were processed through sequential extraction in Tris-buffered saline (TBS), followed by 1% Triton-X-100 in TBS (TBS-X), and finally 5M guanidine in 50mM Tris as described (21). Aβ_40_ and Aβ_42_ levels were determined by enzyme-linked immunosorbent assay (ELISA) as previously described (21). Antibodies, ELISAs, and mammalian-produced recombinant proteins for CLU and PLXNA4 were from R & D Systems.

### Immunoprecipitation of CLU protein from mouse brain

WT and *Clu^-/-^* mice were transcardially perfused with phosphate-buffered saline (PBS) to flush the cerebrovasculature of blood-derived CLU. All steps were performed on ice or at 4 °C. Brains were lysed in PBS, centrifuged at 20,000 x *g* to pellet PBS-insoluble material (including cell membranes), and the pellet was lysed in 1% Triton-X-100-PBS. The initial detergent-free PBS lysis step was used to remove soluble CLU so as not to titrate antibody away from CLU-receptor complexes. The 1% Triton fraction was immediately diluted 10-fold in PBS (0.1% Triton final), pelleted by centrifugation at 20,000 x *g*, and the supernatant was used to immunoprecipitate (IP) with goat anti-CLU for 1 hour at 4 °C. Complexes were gently pelleted at 1,000*g* with protein-A/G-sepharose beads and washed once with cold PBS. Immunoprecipitated material was boiled in 1X protein sample buffer and loaded onto SDS-PAGE gels followed by silver staining per manufacturers protocols (Pierce).

### Mass spectrometry identification of PLXNA4

Identification of silver-stained gel band was performed by the Proteomics Core at Mayo Clinic. The gel band was destained with 15mM potassium ferricyanide and 50mM sodium thiosulfate in water until clear, then rinsed with water several times to remove all colour. The bands are treated with 50 mM TCEP/50 mM Tris, pH 8.1 at 55ºC for 40 min to reduce cysteines, followed by alkylation with 40mM iodoacetamide/50mM Tris pH 8.1 at room temperature for 40 min in the dark. Proteins are digested in-situ with trypsin (Promega Corporation, Madison WI) in 20 mM Tris pH 8.1/0.0002% Zwittergent 3–16, at 37 °C overnight, followed by peptide extraction with 10µl of 2% trifluoroacetic acid and 60ul acetonitrile. Extracts are concentrated to less than 5ul on a spinning vacuum concentrator and brought up in 0.15% formic acid/0.05% trifluoroacetic acid for protein identification by nano-flow liquid chromatography electrospray tandem mass spectrometry (nanoLC-ESI-MS/MS) using a Thermo Scientific Orbitrap Elite Hybrid Mass Spectrometer (Thermo Fisher Scientific, Bremen, Germany) coupled to an Eksigent nanoLC-2D HPLC system (Eksigent, Dublin, CA). The digest peptide mixture is loaded onto a 250nl OPTI-PAK trap (Optimize Technologies, Oregon City, OR) custom packed with Michrom Magic C8 solid phase (Michrom Bioresources, Auburn, CA). Chromatography is performed using 0.2% formic acid in both the A solvent (98%water/2%acetonitrile) and B solvent (80% acetonitrile/10% isopropanol/10% water), and a 5%B to 50%B gradient over 60 min at 325 nl/min through a hand packed PicoFrit (New Objective, Woburn, MA) 75μm × 200mm column (Michrom Magic C18, 3um). The Orbitrap Elite mass spectrometer experiment is set to perform a FT full scan from 340-1500 m/z with resolution set at 120,000 (at 400m/z), followed by linear ion trap MS/MS scans on the top 10 ions. Dynamic exclusion is set to 1 and selected ions are placed on an exclusion list for 20 s. The lock-mass option is enabled for the FT full scans using the ambient air polydimethylcyclosiloxane (PCM) ion of m/z = 445.120024 or a common phthalate ion m/z = 391.284286 for real-time internal calibration. Tandem mass spectra are converted to MGF files with Proteome Discoverer 1.3 (Thermo Scientific, San Jose CA) All MS/MS samples are analyzed using Mascot (Matrix Science, London, UK; version 2.2.04), Sequest (ThermoFinnigan, San Jose, CA; version 27, rev. 12) and X! Tandem (www.thegpm.org; version 2006.09.15.3). Scaffold (version Scaffold_3_06_05, Proteome Software Inc., Portland, OR) is used to validate MS/MS based peptide and protein identifications. Peptide identifications are accepted if they can be established at greater than 95.0% probability as specified by the Peptide Prophet algorithm. Protein identifications are accepted if they can be established at greater than 95.0% probability and contain at least three identified peptides. Protein probabilities are assigned by the Protein Prophet algorithm.

### Solid-phase binding

All reagents were from R & D Systems unless otherwise indicated. Mammalian-expressed, recombinant human PLXNA4 protein was coated on MaxiSorp ELISA plates (Nunc) overnight in PBS. Non-specific binding was defined by uncoated wells or using equivalent amounts of bovine serum albumin (BSA) or ovalbumin (all were comparably low). Plates were washed 3X in PBS with 0.05% Tween-20 (PBS-T), blocked in 4% BSA-PBS-T for 1 hour at RT, and washed 3X again in PBS-T. Mammalian-expressed, recombinant human CLU protein was first diluted at indicated concentrations in 0.5% BSA-PBS-T in a separate reagent plate. The assay plate was aspirated and CLU was added with a multichannel pipette and incubated for 10 min at 37 °C. The plate was then washed 5X in PBS-T and biotinylated anti-CLU antibody diluted in 0.5% BSA-PBS-T was added and incubated for 1 hour at 37 °C. Plates were washed 5X in PBS-T and horseradish peroxidase conjugated to streptavidin diluted in 0.1% BSA-PBS-T was added and incubated for 30 min at room temperature. Plates were washed 5X in PBS-T and developed with Super Slow TMB substrate for ELISA reagent (Sigma).

### Behavioural analysis

A behavioural battery consisting of open field assay (OFA), elevated plus maze (EPM) test, and contextual and cued fear conditioning (CFC) was performed as described (12,13,22,23). All cohorts used male and female littermates from heterozygous intercrosses of *Plxna4^+/-^* males bred to *Plxna4^+/-^* females to derive each of the genotypes: *Plxna4^+/+ ^*“wild-type” (WT), *Plxna4^+/-^* (heterozygous), and *Plxna4^-/-^* (knockout) mice. The experimenter was blinded to the genotypes. Mice were acclimated to the testing room for one hour prior to testing, and all tests were performed during the first half of the light cycle, except for cued fear conditioning. All behavioural equipment was thoroughly cleaned with 30% ethanol and dried between each animal. Mice were returned to their home cage and homeroom after each test.

### Open-field assay

Mice were placed in the centre of an open-field arena (40 × 40 × 30 cm, W × L × H), and allowed to roam freely for 15 min. An overhead camera was used to track movement with AnyMaze software (Stoelting Co., Wood Dale, IL), and mice were analyzed for multiple measures, including total distance travelled, average speed, time mobile, and distance travelled in an imaginary “centre” zone (20 × 20 cm).

### Elevated plus maze assay

As a formal test of anxiety/exploration, the entire maze is elevated 50cm from the floor, and consists of four arms (50 × 10cm) with two of the arms enclosed with roofless gray walls (35 × 15cm, L × H). Mice were placed in the centre of the maze facing an open arm, and their behaviour was tracked for 5 min with an overhead camera and AnyMaze software.

### Contextual and cued fear conditioning assay

This test was conducted in a sound attenuated chamber with a grid floor capable of delivering an electric shock and freezing was measured with an overhead camera and FreezeFrame software (Actimetrics, Wilmette, IL). Mice were initially placed into the chamber and left undisturbed for 2 min to record baseline freezing behaviour. An 80-dB white noise served as the conditioned stimulus (CS) and was presented for 30 sec. During the final 2 sec of this noise, mice received a mild foot shock (0.5mA), which served as the unconditioned stimulus (US). After 1 min, another CS-US pair was presented. The mouse was removed 30 sec after the second CS-US pair and returned to its home cage. Twenty-four hours later, each mouse was returned to the test chamber and freezing behaviour was recorded for 5 min (context test dependent upon hippocampus function). Mice were returned to their home cage and placed in a different room than previously tested in reduced lighting conditions for a period of no less than one hour. For the auditory CS test, environmental and contextual cues were changed by: wiping testing boxes with 30% isopropyl alcohol instead of 30% ethanol; replacing white house lights with red house lights; placing a coloured plastic triangular insert in the chamber to alter its shape and spatial cues; covering the wire grid floor with opaque plastic; and altering the chamber scent with vanilla extract. The animals were placed in the apparatus for 3 min and then the auditory CS was presented and freezing was recorded for another 3 min (cued test dependent upon amygdala and hippocampus function). Baseline freezing behaviour obtained during training was subtracted from the context or cued tests to control for animal variability. No baseline freezing differences among the genotypes were found for this assay.

### Statistical analyses

Data were analyzed by unpaired two-tailed t tests for two groups or ANOVA with post-hoc t-tests for three groups in GraphPad Prism. All data is presented as mean +/- SEM. p < 0.05 was considered statistically significant.

## Supplementary Material

Supplementary Material is available at *HMG* online.

Supplementary Data
