# Supplementary material for: Identification of plexin A4 as a novel clusterin receptor links two Alzheimer’s disease risk genes
Source: Hum Mol Genet. 2016 Jul 4;25(16):3467–75. doi: 10.1093/hmg/ddw188 (PMC5179943; doi:10.1093/hmg/ddw188)
Supplement: Supplementary Data [file supp_25_16_3467__index.html]

Identification of plexin A4 as a novel clusterin receptor links two Alzheimer’s disease risk genes — Identification of plexin A4 as a novel clusterin receptor links two Alzheimer’s disease risk genes — Supplementary Data 

# Identification of plexin A4 as a novel clusterin receptor links two Alzheimer’s disease risk genes

## Supplementary Data

files

- Supplementary Data - docx file
