# Supplementary material for: Identification of plexin A4 as a novel clusterin receptor links two Alzheimer’s disease risk genes
Source: Hum Mol Genet. 2016 Jul 4;25(16):3467–75. doi: 10.1093/hmg/ddw188 (PMC5179943; doi:10.1093/hmg/ddw188)
Supplement: Supplementary Data [file supp_ddw188_Kang.Fryer.PLXNA4.HMG.Supp.docx]

**SUPPLEMENTAL DATA**

**Supplemental Figure 1. Validation of PLXNA4 antibodies by Western blotting.** *Plxna4^+/+^* (WT), *Plxna4^+/-^* and *Plxna4^-/-^* adult brain tissue lysates were probed by Western blotting with three different commercial antibodies to determine specificity. All three antibodies gave robust signal in WT mice, ~50% reduction in *Plxna4^+/-^* lysates, and no signal in *Plxna4^-/-^* lysates.

**Supplemental Figure 2. Baseline freezing in the contextual fear conditioning task is not altered by reduction or absence of PLXNA4.** A) Freezing behavior was not significantly different among the genotypes during the training phase of the contextual fear conditioning task (first two minutes before presentation of tone or foot shock). B) Freezing behavior was also not significantly different among the genotypes during the first three minutes of the cued phase of the contextual fear conditioning task (before presentation of the tone). Data analyzed by one-way ANOVA. N=22/genotype of males and females combined.
